# Shear wave dispersion to assess liver disease progression in Fontan-associated liver disease

**DOI:** 10.1371/journal.pone.0271223

**Published:** 2022-07-08

**Authors:** Tomoaki Nagasawa, Hidekatsu Kuroda, Tamami Abe, Hirofumi Saiki, Yasuhiro Takikawa

**Affiliations:** 1 Division of Hepatology, Department of Internal Medicine, Iwate Medical University, Yahaba-cho, Shiwa-gun, Iwate, Japan; 2 Department of Pediatrics, Iwate Medical University, Yahaba-cho, Shiwa-gun, Iwate, Japan; Medizinische Fakultat der RWTH Aachen, GERMANY

## Abstract

**Aim:**

We aimed to analyze the dispersion slope (DS) using shear wave dispersion (SWD) in patients with Fontan-associated liver disease (FALD) and to investigate its utility as a biomarker of disease progression.

**Methods:**

This cross-sectional study enrolled 27 adults with FALD who underwent SWD, two-dimensional shear wave elastography (2D-SWE), transthoracic echocardiography, cardiac catheterization, or abdominal computed tomography (CT) from April 2019 to April 2021. According to CT findings, patients were divided into two groups: significant fibrosis and non-significant fibrosis.

**Results:**

The median DS in the control (n = 10), non-significant fibrosis (n = 12), and significant fibrosis (n = 15) was 9.35, 12.55, and 17.64 (m/s)/kHz, respectively. The significant fibrosis group showed a significantly higher DS than non-significant fibrosis group (P = 0.003). DS showed a significant correlation with central venous pressure (r = 0.532, P = 0.017) and liver stiffness measurements using 2D-SWE (r = 0.581, P = 0.002). The areas under the receiver operating characteristic curve for the diagnosis of significant fibrosis were 0.903 and 0.734 for SWD and 2D-SWE, respectively (P = 0.043).

**Conclusions:**

DS measured by SWD reflects the severity of liver damage in patients with FALD. SWE may be valuable for the therapeutic management of patients with FALD.

## Introduction

There has been an improvement in the survival of patients with complex congenital heart disease due to successful reparative surgeries, including the Fontan procedure. This procedure is considered the definitive palliation for patients with single-ventricle physiology [1, 2]. The Fontan procedure involves anastomosis between the vena cava or right atrium and the pulmonary arteries, which allows systemic venous blood to bypass a pumping chamber when returning to the lungs [1, 3]. However, the hemodynamic characteristics of Fontan circulation, involving passive circulation of blood from the central vein to pulmonary artery, result in relatively high central venous pressure (CVP), impaired structural and functional properties of the peripheral vessels, impaired cardiac function, and depressed cardiac output. These unusual hemodynamics have led to the concerns about the long-term effects of the Fontan circulation becoming increasingly apparent, along with many distant complications being reported [4, 5]. In particular, the incidence of a hepatic complication known as Fontan-associated liver disease (FALD) is increasing [5, 6]. In FALD, the underlying mechanism of liver injury is persistent chronic passive sinusoidal congestion. In a study of 241 patients with FALD, cirrhosis was observed in approximately one-third of the patients [7]. Cirrhosis may eventually progress to hepatocellular carcinoma. Therefore, implementing evidence-based monitoring and management strategies is essential for patients who have undergone the Fontan procedure. Further, the accurate detection and grading of hepatic fibrosis is important in estimating the risk of developing cirrhosis and hepatocellular carcinoma as well as prognosis in patients who have undergone the Fontan procedure [8, 9]. Liver biopsy is the gold standard for the diagnosis of FALD. However, liver biopsy is invasive and is prone to sampling errors as well as intra- and inter-observer variability [9, 10]. Moreover, liver biopsy is frequently contraindicated in patients with FALD because they often take anticoagulant agents. Most patients with FALD have been reported to have abnormal findings on diagnostic imaging. FALD cases with significant fibrosis have uneven liver surface nodularity, liver reticular enhancement, and portosystemic collateral circulation on computed tomography (CT) [9, 11, 12]. Recently, there have been some reports on liver stiffness measurement (LSM) using ultrasound elastography. Two-dimensional shear wave elastography (2D-SWE) is a type of ultrasound elastography that uses shear waves for assessment of tissue elasticity and viscosity and quantitative display [13, 14]. 2D-SWE can measure shear wave elasticity, which is related to LSM. While ultrasound elastography is commonly used to detect and measure hepatic fibrosis, its use in patients following Fontan palliation can be more challenging [15, 16]. This is because both congestion and fibrosis cause liver stiffening. Previous reports have shown that liver stiffness is a confounding biomarker, with considerable liver stiffening being present and persisting immediately following completion of the Fontan procedure [16, 17].

On the other hand, there is evidence that shear wave dispersion (SWD) is related to the frequency dependence of both velocity and attenuation of the shear wave of the viscous component; as the frequency of the dispersed structure increases, both velocity and attenuation of the shear wave become more pronounced [18, 19]. Thus, the liver viscosity could be measured indirectly using the dispersion slope (DS) calculated using SWD. Previous studies have shown that Fontan circulation predisposes patients to chronic hepatic passive congestion and necrosis, which may progress to hepatic fibrosis [9, 19, 20]. Progression of liver fibrosis has been reported to increase liver elasticity, and DS has been reported to be associated with inflammation, necrosis, steatosis, and congestion [20, 21]. However, the clinical implications of 2D-SWE have not been well investigated in patients with FALD. Further, the clinical significance of SWD remains unknown.

Therefore, we hypothesized that the DS measured using SWD, which reflects liver viscosity, is sensitively elevated in FALD, reflecting hepatic congestion. Furthermore, it may be helpful to clarify the progression of liver disease in patients with FALD. This study aimed to evaluate the efficacy of SWD in assessing the progression of liver disease in patients with FALD and to directly compare the diagnostic ability of 2D-SWE performed on the same day.

## Materials and methods

### Patients

This cross-sectional study included patients evaluated at the Iwate Medical University Hospital, Iwate, Japan. In this study, consecutive patients included 30 adults with FALD who underwent SWD, 2D-SWE, transthoracic echocardiography (TTE), cardiac catheterization, and abdominal computed tomography (CT) from April 2019 to April 2021. The inclusion criteria were the ability to provide informed consent and age between 10 and 50 years. The exclusion criteria included the following: alcohol use (consuming ≥40 g alcohol/day for men and ≥20 g alcohol/day for women in the preceding 12 months) and other liver diseases, such as viral liver disease and autoimmune hepatitis. The control group comprised 10 age- and sex-matched participants with normal liver enzyme levels. All procedures followed were in accordance with the ethical standards of the responsible committee on human experimentation (institutional and national) and with the principles of the Helsinki Declaration. This study was approved by the Ethics Review Board of Iwate Medical University (MH2019–177). Prior to the start of the study, written informed consent was obtained from all controls and patients to use their laboratory data.

### 2D-SWE and SWD

All 2D-SWE and SWD examinations were performed with a diagnostic US scanner (Aplio i800; Canon Medical Systems Corp., Otawara, Tochigi, Japan) with a 3.5-MHz convex transducer (PVI-475BX) by one of two experienced radiologists with 15 years of experience with liver US. The radiologists were blinded to the patients’ clinical data. Patients were placed in the supine position with their right upper extremity lifted; in addition, patients were under fasting for 4 h. The liver target area was placed under the guidance of a conventional, real-time, B-mode image. Scanning was performed between the ribs in the right lobe of the liver (segment 5) with minimal scanning pressure. A sample box measuring approximately 30 × 30 mm was placed on a B-mode image at least 10 mm beneath the liver capsule to avoid reverberation artifacts. After evaluating the shear wave propagation by the emission of an acoustic radiation impulse, data were acquired in the sample box. One LSM was obtained from each 2D-SWE image. The median value of 10 LSMs was used to represent liver stiffness.

**Fig 1:**
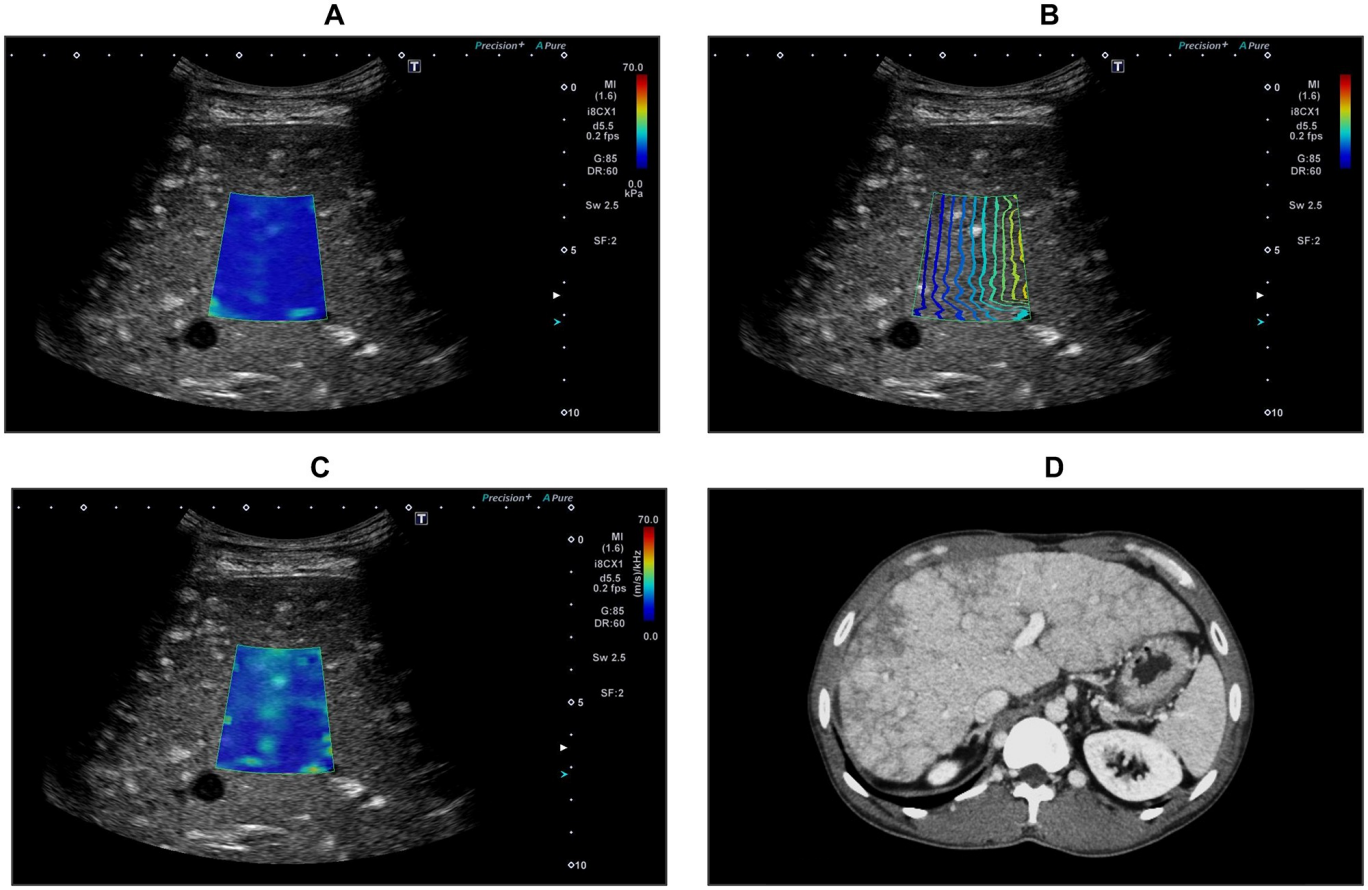
Two-dimensional shear wave elastography (2D-SWE), shear wave dispersion (SWD), and abdominal computed tomography (CT) images. 2D-SWE color map (a), propagation map (b), SWD color map (c), and abdominal CT (d) in patients with FALD.

The DS was analyzed using SWD according to the technique reported in a previous study [19, 21, 22]. In this study, the ultrasonic system automatically displayed the quad view mode, including a 2D-SWE color map (Fig 1a), propagation map (Fig 1b), B-mode image, and SWD color map (Fig 1c). As a result, shear wave elasticity and DS data can be observed simultaneously. The SWD map obtains shear wave velocity and analyzes shear wave frequency, and then calculates the slope of the shear wave velocity versus frequency dispersion of the shear wave. The viscosity of the tissue was measured using this new map. The median value of 10 DS values was used to represent liver viscosity. DS values are expressed as (meter/second)/kilohertz. After recording all DS measurements, data were screened based on the following criteria: ≥10 valid measurements, 60% success rate (ratio of valid acquisitions to total acquisitions), and interquartile range of <30% of the median DS.

### TTE and Pulsed-Wave Doppler (PWD) ultrasound

TTE was performed within 6 months of SWD measurements. The left ventricular ejection fraction, the maximal diameter of the inferior vena cava during expiration [IVC diameter (exp)], the minimal diameter of the IVC during inspiration [IVC diameter (insp)], and the collapsibility index of IVC were measured. We recorded hepatic vein (HV) waveforms using PWD ultrasound devices simultaneously during SWD measurements. According to previous reports, HV waveforms were visually classified into five types by agreement between the two examiners who were blinded to the clinical data [23, 24]. Moreover, portal vein (PV) velocity and right hepatic artery (HA) velocity were measured using PWD ultrasound.

### Laboratory tests, cardiac catheterization, and CT

Laboratory tests were performed, including hematological tests, biochemical tests, coagulation tests, and measurement of serum fibrosis markers (type 4 collagen-7s and Mac-2-binding protein) simultaneously as the SWD measurements.

Cardiac catheterization was performed within 5 years of SWD measurement. CVP, pulmonary to systemic blood flow ratio, and pulmonary artery resistance to systemic artery resistance ratio (Rp/Rs) were measured in all patients with FALD who underwent hemodynamic study by cardiac catheterization for various indications at our institution.

All patients underwent abdominal CT within 6 months from the date of SWD measurements (Fig 1d). According to previous reports, patients with contrast-enhanced CT findings of liver surface nodularity, the reticular enhancement of the liver, and gastroesophageal varices were classified as the significant fibrosis group, and the remaining patients were classified as the non-significant fibrosis group [9, 11, 12].

### Statistical analysis

Statistical analyses were performed using SPSS (version 23, IBM, Armonk, NY, USA). The values are shown as mean ± standard deviation or median (25^*th*^–75^*th*^ percentile) according to the distribution of the values. Inter-observer and intra-observer agreements were evaluated using the intraclass correlation coefficient (ICC) for the DS values. During comparison the non-significant fibrosis and significant fibrosis groups, Student’s t-test or Mann–Whitney U-test was performed to compare continuous data and the *χ*^2^ test was performed to compare categorical data. Correlations between DS or LSM and other parameters were assessed using Spearman’s rank correlation coefficient. Receiver operating characteristic (ROC) curves were constructed, and the trapezoidal rule calculated the area under the ROC curve (AUROC). Differences in SWD and 2D-SWE diagnostic accuracy were investigated by comparing the AUCs. The optimal cut-off points for predicting different grades of fibrosis were identified based on the highest Youden index. The sensitivity, specificity, positive predictive value (PPV), and negative predictive value (NPV) were calculated using cutoffs obtained from the ROC curves. Statistical significance (P) was set at <0.05.

## Results

### Clinical characteristics of control and patients with FALD

A total of 30 consecutive patients with FALD were screened during the study period. Of these patients, two were excluded due to lack of data on TTE or cardiac catheterization. The success rate for SWD was 96.7% (29/30). The reason for failure with SWD was the inability to perform breath hold optimally (n = 1). Ultimately, 27 patients (90.0%) were included in the statistical analysis. Liver surface nodularity, reticular enhancement of the liver, and gastroesophageal varices were observed in 44.4% (12/27), 37.0% (10/27), and 51.9% (14/27) of patients, respectively. Finally, all patients were classified into the significant fibrosis group (n = 15) and the non-significant fibrosis group (n = 12).

The characteristics of overall subjects are summarized in Table 1. At the time of evaluation, the age of patients was 19.9 ± 6.2 years in the non-significant fibrosis group and 23.8 ± 11.9 years in the significant fibrosis group. The mean time elapsed since the Fontan procedure in the non-significant fibrosis and significant fibrosis groups was 16.2 ± 6.7 and 19.9 ± 9.4 years, respectively, with the significant fibrosis group showing a significantly longer time elapsed (P = 0.041). No parameters were found to be statistically significant between the two groups in hematological tests, biochemical tests, coagulation tests, and serum fibrosis markers.

**Table 1:** Clinical characteristics of control and FALD patients.

Variables	control	Non-significant fibrosis	Significant fibrosis
No. of patients	10	12	15
Sex (male/female)	4/6	4/8	7/8
Mean age (years)	20.3±3.1	19.9±6.2	23.8±11.9
BMI (kg/m2)	20[18.3–20.8]	19.9[17.7–24.2]	20.9[18.5–22.3]
Times elapsed the Fontan procedure (years)		16.2±6.7	19.9±9.4^†^
T.Bil (mg/dL)	0.9[0.7–1.1]	0.9[0.7–1.0]	1.0[0.9–1.2]
AST (U/L)	25.0[21.5–30.5]	22.0[20.8–26.5]	23.0[20.5–32.5]
ALT (U/L)	23.0[22.0–29.5]	22.0[17.0–37.5]	21.0[17.5–27.5]
Alb (g/dL)	4.5[4.2–4.8]	4.4[4.2–4.6]	4.3[4.2–4.6]
GGT (U/L)	54.5[32.5–56.5]	62.0[33.5–90.5]	95.5[62.5–136.7]*
ALP (U/L)	195.5[162.5–232.5]	230.5[203.8–672.0]	266.5[213.7–352.0]
TC (mg/dL)	183.5[171.5–196.0]	155.5[124.0–175.0]	143.5[138.0–162.5]*
TG (mg/dL)	89.0[54.5–117.0]	83.0[54.5–118.8]	63.0[51.0–71.0]
Hb (g/dL)	14.0[13.3–15.8]	15.1[13.8–15.7]	14.5[13.9–16.7]
Plt (×104/mm3)	28.5[24.8–30.5]	22.5[20.3–28.9]*	19.9[13.7–25.7]*
PT-INR	1.0[0.9–1.1]	1.3[1.2–1.4]*	1.3[1.2–1.5]*
AFP (ng/mL)	1.8[1.4–3.3]	1.8[1.5–3.6]	1.9[1.1–3.6]
Type IV collagen-7S (ng/mL)	3.0[2.0–3.8]	5.9[5.4–7.1]*	6.9[6.3–7.3]*
M2BPGi (COI)	0.3[0.2–0.4]	0.5[0.4–0.6]**	0.6[0.3–0.7]**
BNP (pg/mL)	10.5[9.3–12.5]	16.2[7.6–28.5]	31.1[9.6−72.9]*
FIB-4 index	0.3[0.3–0.4]	0.5[0.3–0.7]**	0.6[0.5–0.7]*

The values represent the mean ± standard deviation, the median [25–75th percentile], or number of patients.

* P<0.01 (compared with control).

** P<0.05 (compared with control).

^†^ P<0.05 (compared with Non-significant fibrosis group).

Abbreviations: BMI, body mass index; T.Bil, total bilirubin; AST, aspartate aminotransferase; ALT, alanine aminotransferase; Alb, albumin; GGT, gamma-glutamyl transferase; ALP, alkaline phosphatase; TC, total cholesterol; TG, triglyceride; Hb, hemoglobin; Plt, platelet; PT, prothrombin time; INR, international normalized ratio; AFP, alpha-Fetoprotein; M2BPGi, Mac-2 binding protein glycan isomer; BNP, brain natriuretic hormone.

The major diagnoses and types of Fontan procedure are presented in Table 2. The Fontan types included atriopulmonary connection in one (3.7%) patient and total cavopulmonary connection in 23 (85.2%) patients, five of whom were converted from atriopulmonary connection. Five patients (19.5%) had open fenestration in the Fontan conduit.

**Table 2:** Diagnosis and type of Fontan procedure in FALD patients.

Major disease	n	Type of Fontan procedure	n
DIRV	7	TCPC	13
TA	5	TCPC with fenestration	5
PA/IVS	2	TCPC converted from APC	5
Unbalanced AVSD	2	APC	1
DILV	2	others	3
HLHS	1		
Others	8		

Abbreviations: PA/IVS, pulmonary atresia with intact ventricular septum; AVSD, atrioventricular septal defect; TA, tricuspid atresia; HLHS, hypoplastic left heart syndrome; DIRV, double inlet right ventricle; DILV, double inlet left ventricle; TCPC, total cavopulmonary connection; APC, atriopulmonary connection

### Inter-observer and intra-observer reliability of SWD

The ICC for intra-observer agreement of DS measurements using SWD was 0.86 [95% confidence interval (CI): 0.76–0.95]. Furthermore, the reproducibility of SWD between observers (for all patients) yielded an ICC of 0.81 (95% CI: 0.71–0.91).

### Association of DS and LSM with different parameters

**Fig 2:**
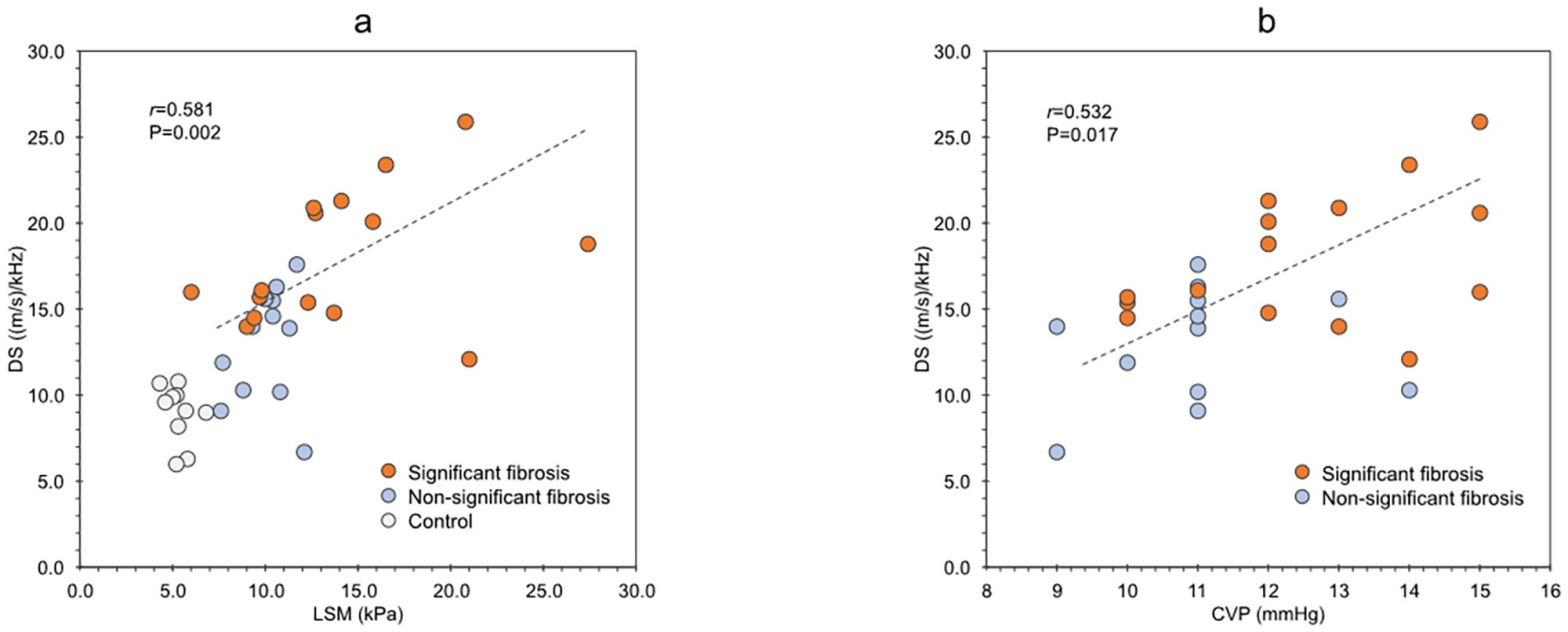
Relationship between the dispersion slope (DS) and liver stiffness measurement (LSM) in the control group (n = 10) and patients with FALD (n = 27). The correlation efficient (r) and P-value were calculated using Spearman’s rank correlation coefficient (a). Relationship between the dispersion slope (DS) and central venous pressure (CVP) in the control group (n = 10) and patients with FALD (n = 27) (b). DS, dispersion slope; LSM, liver stiffness measurements; CVP, central venous pressure.

CVP was the only parameter that showed a significant correlation with DS (r = 0.532, P = 0.017), whereas no parameter showed a significant correlation with LSM (Table 3). Further, DS showed a significant correlation with LSM (r = 0.581, P = 0.002) (Fig 2).

**Table 3:** Correlation between DS or LSM and different parameters in Fontan patients.

Variables	DS ((m/s)/kHz)		LSM (kPa)	
	r	P-value	r	P-value
Age (years)	-0.245	0.218	-0.082	0.685
BMI (kg/m2)	-0.196	0.326	-0.063	0.754
Times elapsed the Fontan procedure (years)	0.335	0.077	0.316	0.167
T.Bil (mg/dL)	0.021	0.918	0.195	0.329
AST (U/L)	0.091	0.650	0.255	0.199
ALT (U/L)	0.198	0.321	0.144	0.472
Alb (g/dL)	0.207	0.300	0.342	0.081
GGT (U/L)	0.249	0.458	0.138	0.490
ALP (U/L)	0.164	0.218	0.068	0.737
TC (mg/dL)	-0.312	0.154	-0.278	0.161
TG (mg/dL)	-0.346	0.078	-0.180	0.368
Hb (g/dL)	-0.025	0.901	-0.012	0.954
Plt (×104/mm3)	-0.158	0.430	-0.305	0.122
PT-INR	0.042	0.835	0.026	0.898
AFP (ng/mL)	-0.023	0.910	-0.177	0.376
Type IV collagen-7S (ng/mL)	0.265	0.182	0.158	0.431
M2BPGi (COI)	0.271	0.170	0.284	0.150
BNP (pg/mL)	0.054	0.789	0.212	0.288
FIB-4 index	0.016	0.938	0.308	0.118
Vmax (HA) (m/s)	0.176	0.623	0.116	0.811
Vmax (PV) (m/s)	-0.141	0.509	-0.049	0.820
LVEF (%)	0.176	0.347	0.271	0.398
IVC diameter (exp) (cm)	0.275	0.241	0.312	0.549
IVC diameter (insp) (cm)	0.059	0.781	0.070	0.524
IVC collapsibility index	0.287	0.295	0.287	0.140
CVP (mmHg)	0.532	0.017	0.311	0.164
Qp/Qs	-0.355	0.162	-0.211	0.295
Rp/Rs	-0.068	0.615	-0.029	0.780

Abbreviations: DS, Dispersion Slope; LSM, Liver stiffness measurement; BMI, body mass index; T.Bil, total bilirubin; AST, aspartate aminotransferase; ALT, alanine aminotransferase; Alb, albumin; GGT, gamma-glutamyl transferase; ALP, alkaline phosphatase; TC, total cholesterol; TG, triglyceride; Hb, hemoglobin; Plt, platelet; PT, prothrombin time; INR, international normalized ratio; AFP, alpha-Fetoprotein; M2BPGi, Mac-2 binding protein glycan isomer; BNP, brain natriuretic hormone; Vmax: peak systolic flow velocity; HA, hepatic artery; PV, portal vein; LVEF, left ventricular ejection fraction; IVC, inferior vena cava; exp, expiration; insp, inspiration; CVP, central venous pressure; Qp/Qs, pulmonary to systemic flow ratio; Rp/Rs, pulmonary artery resistance/systemic artery resistance ratio

### Association of DS and postoperative years

The entire FALD group was classified by age group (<10 years, 10–15 years, and 15<years) as years after Fontan procedure, and DS was compared by age group, DS increased with years since surgery, but the difference was not statistically significant (S1 Fig).

### Ultrasound and hemodynamic parameters in the patient groups

**Fig 3:**
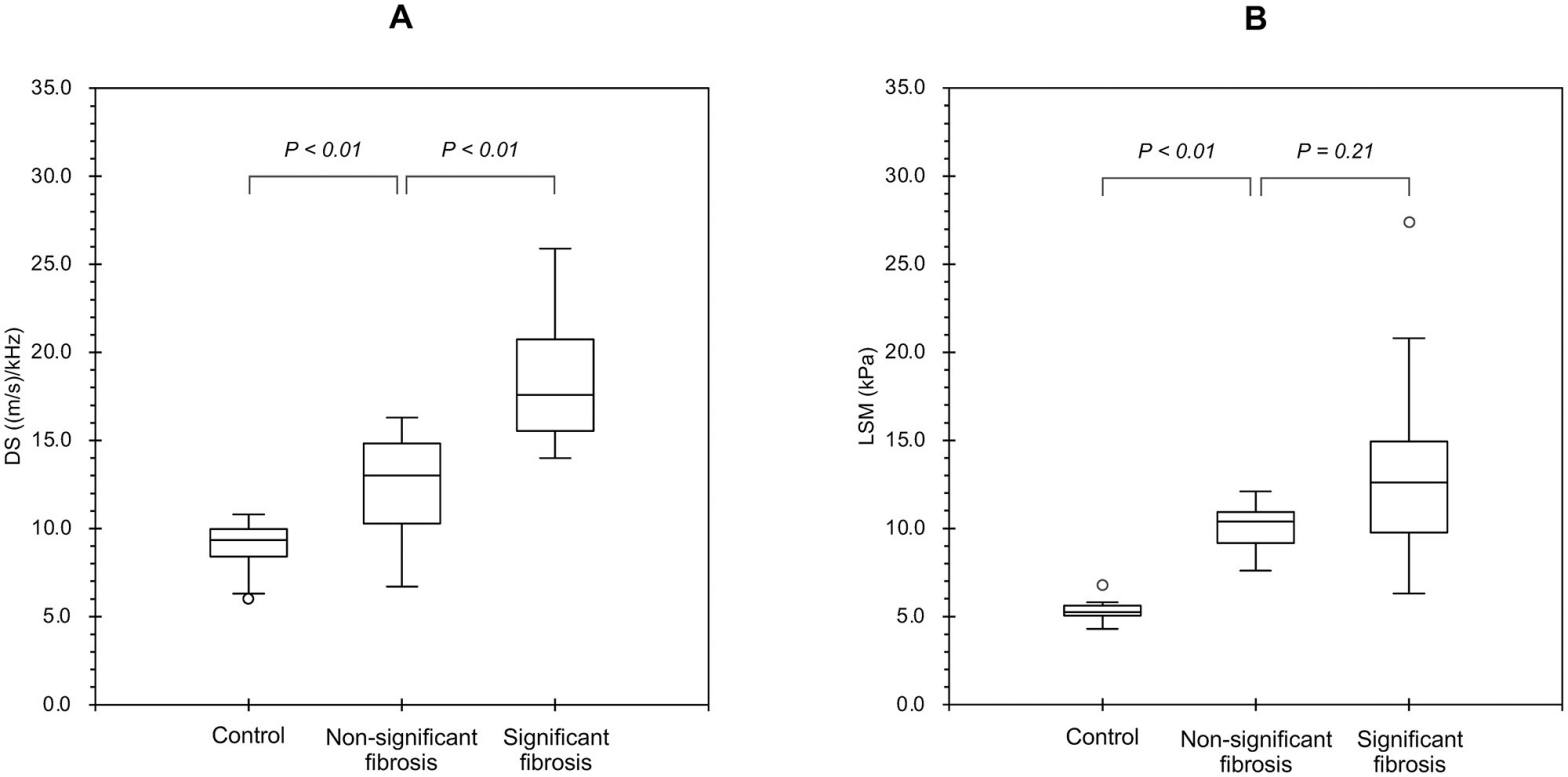
DS and LSM distribution for different groups. The median DS in the control, non-significant fibrosis, and significant fibrosis groups was 9.35, 12.55, and 17.64 (m/s)/kHz, respectively (a). The significant fibrosis group showed significantly higher DS than the non-significant fibrosis group. The median LSM in the control, non-significant fibrosis, and significant fibrosis groups were 5.05, 10.80, and 12.60 kPa, respectively (b). There were no significant differences in LSM between the non-significant fibrosis and significant fibrosis groups.

Table 4 shows the comparison of the ultrasound and hemodynamic parameters between the non-significant fibrosis and significant fibrosis groups. Only DS showed a significant difference between the two groups. The median DS in the control, non-significant fibrosis, and significant fibrosis groups was 9.35, 12.55, and 17.64 (m/s)/kHz, respectively (Fig 3a). The significant fibrosis group showed a significantly higher DS than the non-significant fibrosis group (P = 0.003). On the other hand, the median LSM in the control, non-significant fibrosis, and significant fibrosis groups was 5.05, 10.80, and 12.60 kPa, respectively (Fig 3b). There were no significant differences in LSM between the non-significant fibrosis and significant fibrosis groups.

**Table 4:** The ultrasound and hemodynamic parameters in non-significant fibrosis and significant fibrosis.

Variables	Non-significant fibrosis	Significant fibrosis	P value
LSM (kPa)	10.80[9.18–10.92]	12.60[9.75–14.95]	0.216
DS ((m/s)/kHz)	12.55[10.27–14.85]	17.64[15.51–20.70]	0.003
Vmax (HA) (m/s)	46.20[37.8–50.6]	58.30[41.5–75.6]	0.250
Vmax (PV) (m/s)	17.50[13.6–21.6]	16.50[12.6–19.0]	0.763
HV waveform (type 1/2/3/4/5)	3/4/3/1/1	0/4/6/3/2	0.281
Liver tumor (+/-)	1/11	4/11	0.223
HES (+/-)	4/8	8/7	0.299
LVEF (%)	56.900[52.8–59.4]	57.700[53.0–61.6]	0.622
IVC diameter (exp) (cm)	1.470[1.19–1.79]	1.490[1.31–1.94]	0.895
IVC diameter (insp) (cm)	0.960[0.76–1.28]	0.940[0.72–1.19]	0.914
IVC collapsibility index	28.713[27.8–41.1]	37.626[31.5–49.7]	0.334
CVP (mmHg)	11.0[10.0–13.0]	13.0[12.0–15.0]	0.058
Qp/Qs	1.000[0.937–1.000]	0.936[0.843–0.953]	0.569
Rp/Rs	0.086[0.062–0.112]	0.075[0.057–0.082]	0.970

Abbreviations: LSM, Liver stiffness measurement; DS, Dispersion Slope, Vmax: peak systolic flow velocity; HA, hepatic artery; PV, portal vein; HV, hepatic vein; HES, hyperechoic spots; LVEF, left ventricular ejection fraction; IVC, inferior vena cava; exp, expiration; insp, inspiration; CVP, central venous pressure; Qp/Qs, pulmonary to systemic flow ratio; Rp/Rs, pulmonary artery resistance/systemic artery resistance ratio

### Performance characteristics of SWD and 2D-SWE for predicting significant fibrosis

**Fig 4:**
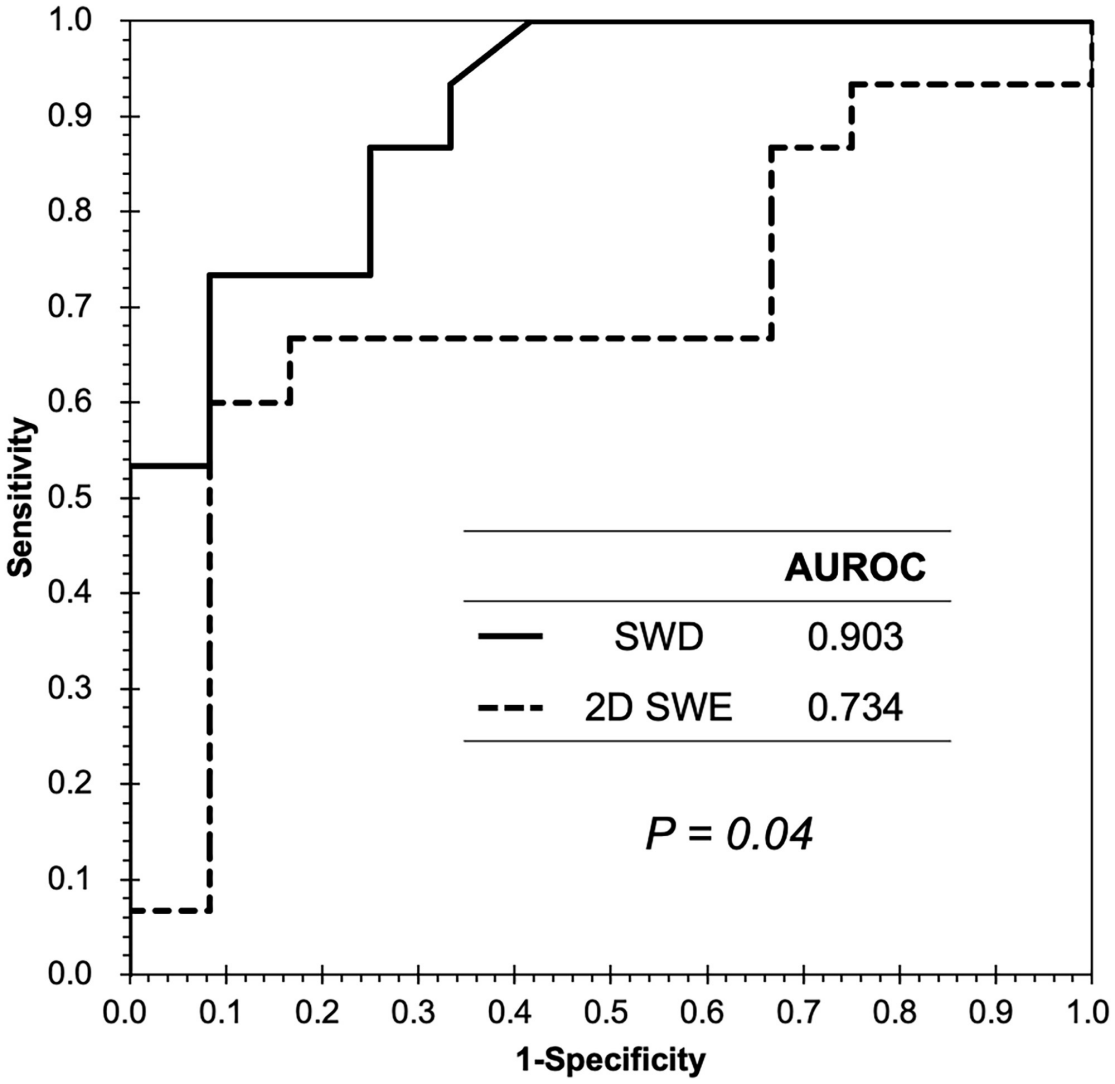
Receiver operating characteristic curve for SWD and 2D-SWE in the diagnosis of significant fibrosis. The AUROC for diagnosing significant fibrosis was 0.903 and 0.734 for SWD and 2D-SWE, respectively. The differences between SWD and 2D-SWE were significant. AUROC, area under the receiver operating characteristic curve; SWD, shear wave dispersion; 2D-SWE, two-dimensional shear wave elastography.

The AUROC for the diagnosis of significant fibrosis was 0.903 and 0.734 for SWD and 2D-SWE, respectively (Fig 4), with significant differences between SWD and 2D-SWE (P = 0.043). The most appropriate cut-off value for SWD in predicting significant fibrosis was 15.60 (m/s)/kHz and the sensitivity, specificity, PPV, and NPV were 73.3%, 91.7%, 91.7%, and 73.3%, respectively.

## Discussion

In this study, we measured the liver viscosity using SWD in 27 patients with FALD and investigated the usefulness of SWD as an indicator of liver disease progression. The results of the study confirmed that there was a characteristic change in liver viscosity in patients with FALD. In these patients, DS (real-timenon-invasive indicator) can reveal tissue damage, especially fibrosis and viscosity. To the best of our knowledge, this is the first cohort study to evaluate the diagnostic ability of SWD in patients with FALD.

The significant pathophysiological finding of FALD is a disturbance in the hepatic blood flow and drainage [6, 9, 25]. Elevated systemic venous pressure in FALD leads to inefficient venous drainage of the liver [9, 25], which is characteristic of chronic passive congestion [9, 25]. The natural history of FALD is reported to have three major stages. The first stage is hepatic congestion and sinusoidal dilation within 10 years after the Fontan procedure. The second stage is hepatic fibrosis without portal hypertension, 10–15 years after surgery. The last stage is advanced fibrosis with portal hypertension, at least 15 years after surgery [25, 26]. Our study included adults with FALD and showed that the significant fibrosis group had a significantly longer time elapsed since the Fontan procedure than the non-significant fibrosis group. The progression of FALD depends on the period of time elapsed after surgery, which is in agreement with the results of previous studies [9, 25].

2D-SWE has been recognized as a valuable method for assessing hepatic fibrosis in chronic liver disease. Liver elasticity significantly increases in cirrhosis [15, 16, 27, 28]. In the field of adult FALD, several studies have reported that liver elasticity is correlated with hepatic fibrosis. However, previous studies have focused only on elasticity using SWE, and assessments of the clinical significance of liver viscosity are lacking. Deorsola et al. reported that congestive hepatopathy affects liver elasticity after the Fontan procedure [16]. Moreover, the usefulness of SWD has not been investigated in patients with FALD. The present study investigated the efficacy of both liver elasticity and viscosity in patients with different severities of hepatic fibrosis among patients with FALD. As a result, this study clarified the gradual changes in liver elasticity and viscosity according to the stage of FALD. Although both DS and LSM elevated in FALD patients, DS more clearly and significantly reflected the severities of hepatic fibrosis than LSM.

The mechanism of liver injury in FALD is suggested to be persistent chronic passive sinusoidal congestion [6, 11, 25]. Elevated CVP and impaired venous drainage cause sinusoidal dilation, and as fibrosis progresses, the central and central veins are cross-linked, leading to cirrhosis. It has also been reported that the severity of fibrosis correlates with both the time elapsed since the Fontan procedure and CVP [9, 11]. In this study, CVP showed a significant correlation with DS, rather than LSM. Based on these findings, DS is susceptible to CVP and may detect hepatic congestion. This study suggests that SWD is sensitive for the diagnosis of liver damage in patients with FALD. Therefore, we recommend that SWD be used for routine follow-up, and we believe it is helpful to use it to detect fibrosis development and to determine when to perform a CT scan.

This study has several limitations. First, the sample size was small. Larger-scale clinical studies are needed to confirm these findings. Second, liver biopsy was not performed for ethical reasons. Third, the histological relationship with DS was not assessed. Fourth, the median interval between cardiac catheterization and abdominal ultrasonography was 3.6 years. The SWD of abdominal ultrasonography may be closely related to hemodynamics, and simultaneous measurements are desirable. Finally, selection bias cannot be ruled out.

In conclusion, the present findings suggest that DS measured by SWD reflects the severity of liver damage in patients with FALD. SWD may be valuable for the therapeutic management of patients with FALD.

## Supporting information

S1 Fig Association of DS and postoperative years. DS distribution for different groups by years after the Fontan procedure. DS increased with the number of postoperative years, but there were no statistically significant differences.
(TIF)
