# Supplementary figures and images for: Shear wave dispersion to assess liver disease progression in Fontan-associated liver disease
Source: PLoS One. 2022 Jul 8;17(7):e0271223. doi: 10.1371/journal.pone.0271223 (PMC9269959; doi:10.1371/journal.pone.0271223)

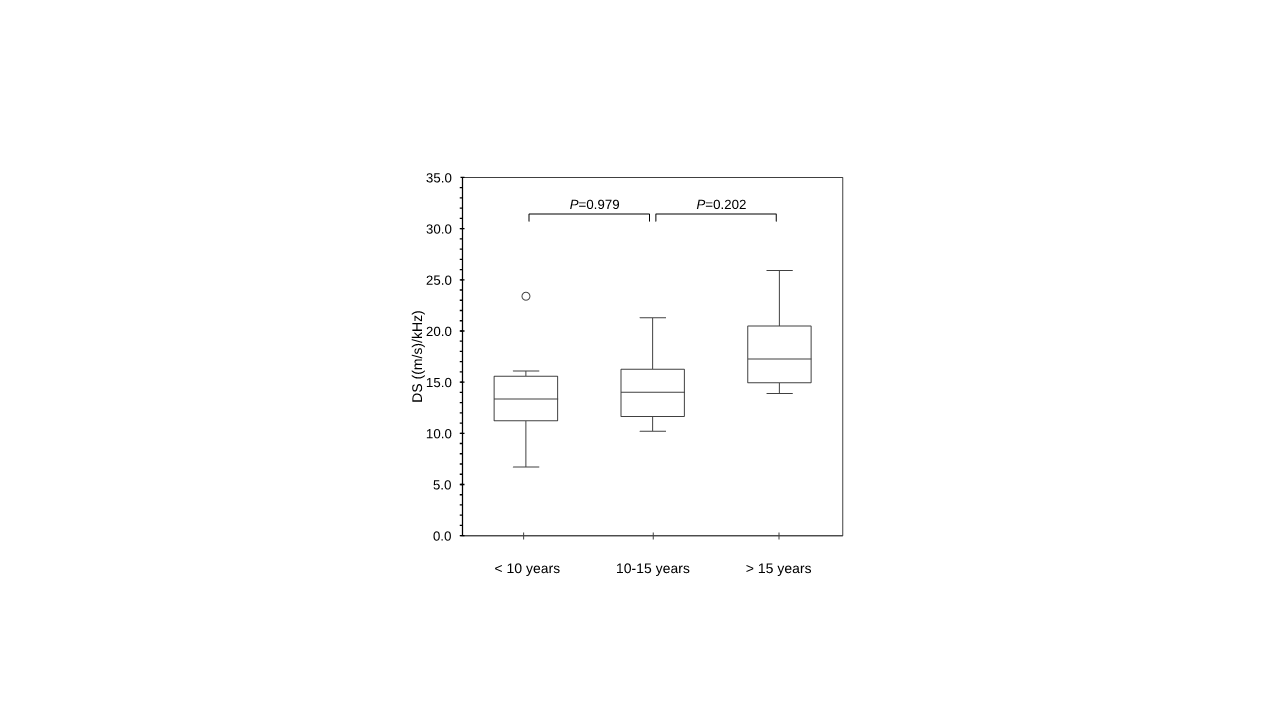

Supplement: S1 Fig — DS distribution for different groups by years after the Fontan procedure. DS increased with the number of postoperative years, but there were no statistically significant differences. (TIF) [file pone.0271223.s001.tif]
